# Etude des aspects cliniques, échographiques et nutritionnels du syndrome des ovaires micropolykystiques (SOMPK) à Mbuji-Mayi, RD du Congo

**DOI:** 10.11604/pamj.2014.19.267.3162

**Published:** 2014-11-11

**Authors:** Louis Mbuyamba Ntobo Kalenda, Justin Biayi Mikenji, Albert Mwembo-Tambwe Nkoy, Prosper Kalenga Muenze Kayamba

**Affiliations:** 1 Département de Gynécologie et Obstétrique, Faculté de Médecine de l'Université Officielle de Mbuji-Mayi, RD du Congo; 2 Département de Gynécologie et Obstétrique, Faculté de Médecine de l'Université de Lubumbashi, RD du Congo; 3 Ecole de Santé Publique de l'Université de Lubumbashi, RD du Congo

**Keywords:** SOMPK, clinique, échographie, nutrition, Mbuji-Mayi, PCOS, clinical, ultrasound, nutrition, Mbuji-Mayi

## Abstract

**Introduction:**

Le Syndrome des Ovaires Micropolykystiques (SOMPK) est polymorphe. L'objectif de cette étude était de déterminer la prévalence et rechercher les facteurs nutritionnels éventuellement associés.

**Méthodes:**

Chez 300 patientes atteintes de SOMPK faisant l'objet de cette étude, les données anamnestiques, cliniques et échographiques, et les habitudes alimentaires ont été analysées. Les statistiques usuelles et les mesures d'association ont été utilisées pour analyser les résultats.

**Résultats:**

La prévalence de SOMPK était de 23,6%. L’âge moyen des patientes avec SOMPK était 24 ± 7 ans et chez celles sans SOMPK 36 ± 7 ans (P < 0,05). Le risque de développer le SOMPK en présence d'un niveau de vie élevé était de 2,03 (RR = 2,03; IC à 95%: 1,73-2,38; P = 0,00). Le risque de développer le SOMPK en exerçant une activité légère est de 2,15 (RR = 2,15; IC à 95%: 1,87-2,87; P= 0,00). La proportion des patientes avec SOMPK ayant accumulé plus de 2400 kcal était de 59% vs 36% (P < 0,05) et le risque de développer le SOMPK lorsque l’énergie accumulée était supérieure à 2400 kcal était de 1,57 (RR = 1,57; IC à 95%: 1,33-1,85; P= 0,00).

**Conclusion:**

Dans notre milieu la prévalence de SOMPK était de 23,6%. Les facteurs associés au SOMPK étaient l’âge, l’énergie accumulée de plus de 2400 Kcal, le niveau socio-économique élevé et l'activité physique légère. Des études analytiques plus poussées sont nécessaires, pour évaluer la force du risque que représentent ces différents facteurs étudiés.

## Introduction

Le syndrome des ovaires micropolykystiques (SOMPK) est une maladie endocrinienne qui affecte les femmes en âge de procréer [1]. Sa fréquence varie selon les études [2]. En Europe, elle varie de 5 à 10% [1, 3]. Alors qu'elle est fort variable en Afrique. Elle a été évaluée à 3% [4], en Côte d'Ivoire, 21% en Afrique du Sud [5] et 22%, en RDC [6]. Ce syndrome associe une spanioménorrhée ou une aménorrhée, une stérilité, un hirsutisme, une obésité, avec des taux sériques anormalement élevés d′androgènes et d′hormone lutéinisante (LH). Une mise en évidence des multiples microkystes ovariens confirme le diagnostic. Un des problèmes à la source des difficultés diagnostiques qui entourent le SOMPK est la fréquence relative des différents signes et symptômes, et le poids qu′il convient d′accorder à l′un plutôt qu′à l′autre dans l′établissement du diagnostic. L'obésité est retrouvée, dans 41% avec une variation de 16 - 44%. Un excès d'apport énergétique avec des rations trop riches en lipides et en glucides à fort index glycémique se déposeraient dans les tissus adipeux et favoriseraient l'obésité. La satisfaction des besoins nutritionnels par une ration alimentaire journalière, raisonnée et utile peut prévenir les excès ou les insuffisances nutritionnelles qui sont néfastes pour la santé [7]. L'objectif de cette étude était de déterminer la prévalence de SOMPK à Mbuji-Mayi et de rechercher ses éventuelles relations avec les facteurs nutritionnels.

## Méthodes

Une étude prospective a été menée pendant une période allant du 1er décembre 2010 au 31 mai 2012 (soit 18 mois), dans quatre centres hospitaliers de Mbuji-Mayi: l'hôpital Dipumba, le centre hospitalier Kayembe, centre hospitalier des Sœurs franciscaines de Saint Esprit de Lukelenge et la polyclinique Notre Dame d'Espérance. La fréquence de SOMPK de 22,2% observée à Kinshasa par Mboloko et coll. en 2004, nous a permis de déterminer la taille de l’échantillon [8] qui été évaluée à 286 patientes et nous avons porté cette taille d’échantillon à 300 pour les patientes avec SOMPK et 300 pour les patientes sans SOMPK. Les patientes avec SOMPK etaient sélectionnées parmi les patientes présentant les signes et les symptômes qui constituent les éléments essentiels du diagnostic de SOMPK et dont l’échographie réalisée avec sonde abdominale connexe de 3,5 MHZ et grâce à l'appareil échographique de marque SONOSITE M. Turbo, a révélé des ovaires micropolykystiques selon les critères du diagnostic échographique recommandé par la conférence de consensus de Rotterdam [9]. Ces patientes avec SOMPK ne recevaient aucun traitement hormonal. Les patientes en âge de procréation sans SOMPK avec les ovaires normaux à l’échographie mais souffrant d'autres maladies (infections, malformations congénitales, prolapsus, viol…) sont recrutées. Celles-ci sont recrutées de manière séquentielle au ratio d'une patiente sans SOMPK pour un cas de SOMPK. A chaque cas a été appariée comme une patiente sans SOMPK la première patiente répondant aux critères définis.

Les paramètres étudiés au moment de l'enregistrement des patientes étaient, à l'anamnèse, l’âge, la parité, la profession de la patiente, l'activité physique exercée et l’évaluation du niveau socio-économique basée sur le score de Traissac et al [10]. L'examen physique recherchait les principaux signes de SOMPK chez les patientes. Il a été complété par l'examen échographique qui a permis de calculer la surface de l'ovaire selon la formule (longueur de l'ovaire) x (largeur de l'ovaire) x (0,8) et le volume des ovaires suivant la formule (longueur de l′ovaire) x (largeur de l′ovaire) x (profondeur de l′ovaire) x (0,523). L'examen échographique a également aidé à compter les nombres de microkystes, à les localiser et à les mesurer. Le grand axe de l'ovaire était aussi mesuré. Les critères de diagnostic échographique des ovaires micropolykystiques est celui recommandé par la conférence de Rotterdam [9].

L'enquête nutritionnelle complétant les données cliniques a consisté à évaluer qualitativement et quantitativement les aliments consommés par chaque patiente. Chaque patiente examinée devait remplir un carnet de consommation de 7 jours en décrivant tous les aliments et boissons consommés à chaque repas et entre les repas. Une estimation de qualité et de quantité a été faite à l'aide d'un carnet de consommation des aliments rempli par la patiente. Elle a été réalisée au niveau de la division du contrôle de qualité des aliments du programme national de nutrition de la RDC [11, 12]. Au cours de la même période, les éclaircissements sur l’état de santé et certaines pratiques alimentaires susceptibles d’être source de risque sanitaire ont été obtenues. L'interrogatoire lors de la remise du carnet des aliments enregistrés nous a permis de connaître les habitudes alimentaires de la patiente et d’établir la ration journalière et d’évaluer les apports énergétiques emmagasinés par chaque malade. En nous basant sur la table de composition des aliments, nous avons dégagé les parts lipidiques, glucidiques et protidiques des aliments consommés [13]. Chaque quantité trouvée était multipliée par sa valeur en calorie: un gramme de lipide donne 9 calories, 1 gramme de glucide donne 4 calories, 1 gramme de protides donne 4 calories et un gramme d'alcool donne 7 calories.

Les critères de jugement et les normes, pour le besoin de l’étude ont retenu la répartition suivante sur la consommation des kilocalories journalières. Les patientes sédentaires qui consommaient 1800 kcal/jour seraient considérées comme celles qui ont un bon apport énergétique journalier, donc ayant un bon état nutritionnel. De même, celles qui exercent une activité physique légère, modérée, intense et qui consommeraient respectivement 2000, 2200 et 2400 kcal par jour seraient considérées comme celles ayant un apport nutritionnel bon, donc avec un état nutritionnel bon. Les patientes qui auraient plus de 2400 kcal consommés par jour et celles ayant 2200 et 2400 Kcal consommés par jour sans exercer l'activité physique en rapport avec ces calories seraient considérées comme celles qui auraient un excès d'apport énergétique donc un état nutritionnel augmenté. Les critères de jugement principal ont portés également sur l'horaire de repas. Nous avons considéré qu'un horaire de trois repas par jour était normal. Sur le plan éthique, la participation à l’étude était libre après un consentement éclairé. Les principes d'anonymat à été respecté. L’étude n'apportait aucune nuisance à la santé des participants.

### Analyse statistique

Pour analyser nos résultats, Nous avons recouru au calcul de la moyenne ± écart type, au calcul de risque relatif (RR), aux tests de X^2^ de Pearson et de Student pour comparer les proportions et les moyennes. L'intervalle de confiance était de 95% avec un seuil de signification fixé à 0,05. Les données ont été encodées et traitées à l'aide des Logiciels Microsoft Excel 2007, Epi Infos 7 et SPSS.

## Résultats

**Figure 1:**
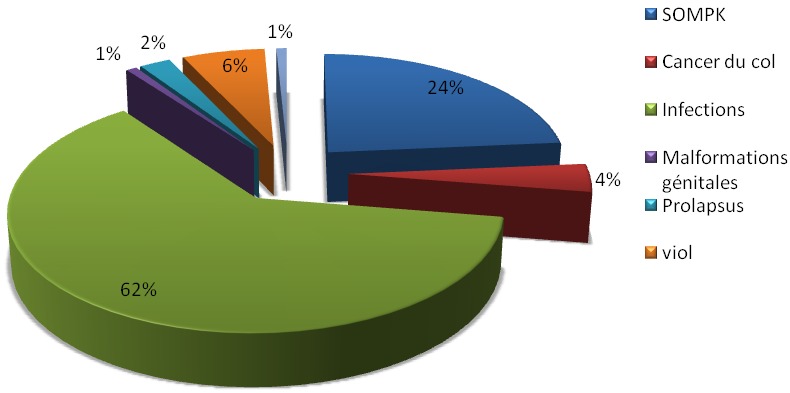
La prévalence des pathologies gynécologiques à Mbuji-Mayi, RDC 2010-2012

**Figure 2:**
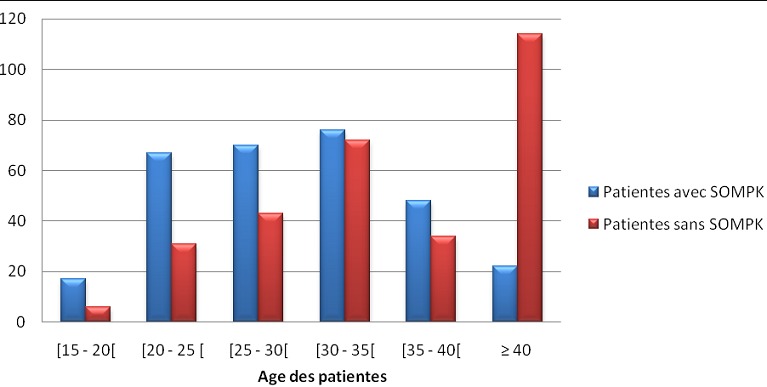
La répartition des patientes selon l’âge (ans)

Nous avons enregistré 300 patientes soit 23,6% qui avaient développé le SOMPK et les 973 autres patientes soit 76,4% qui avaient d'autres pathologies gynécologiques (Figure 1). La prévalence de SOMPK a été évalué à 23,6% alors que les autres pathologies gynécologiques représentées 76,4% (Figure 1). L’âge des patientes avec SOMPK varié entre 15 et 44 ans alors que celui des patientes sans SOMPK va de 15 à 42 ans. L’âge moyen des patientes avec SOMPK est 24 ± 7ans contre 36 ± 7 ans dans le groupe des patientes sans SOMPK (p < 0,05). La proportion des patientes porteuses de SOMPK était prédominante dans la tranche d’âge comprise entre 25 et 35 ans soit 146 cas (48,6%) contre 115 cas (38,3%) dans la même tranche d’âge chez les patientes sans SOMPK. La différence observée était statistiquement significative (p <0,05). Après 40 ans, la proportion de patientes avec SOMPK était moindre (22 cas soit 7,3%) contre une proportion très importante des patientes sans SOMPK (114 cas soit 38%) (Figure 2). Le risque de développer le SOMPK est 1,61 fois plus élevé lorsque la patiente est nullipare (RR = 1,61; IC à 95%:139-1,88), comparé aux autres parités.la parité moyenne des patientes avec SOMPK était 1,7±2,3. Les patientes avec SOMPK ayant comme profession ménagère ou profession libérale sont plus nombreuses par rapport aux patientes sans SOMPK. Les plaintes principales qui ont amené ces patientes à se présenter à la consultation sont l'acné, la stérilité et les troubles du cycle menstruel (spanioménorrhée ou aménorrhée). Nous avons noté que la stérilité a constitué la principale plainte chez les patientes avec SOMPK: Elle a été retrouvée chez 177 patientes avec SOMPK (59%) versus 91 patientes sans SOMPK (30,3%) avec une différence statistiquement significative: Z_cal_= 7,38 > Z_th_ = 1,96; p< 0,05.

Lors de l'examen physique, nous avons noté que l'acanthosis nigrans et l'acné ont été respectivement retrouvé chez 8 (2%) et chez 84 (28%). L'hirsutisme (le score de Ferriman et Gallwey > 8) a été observé uniquement chez 208 patientes (69,3%) avec SOMPK. L'obésité (IMC > à 30) a été observé chez 156 patientes avec SOMPK (52%) versus 54 (18%) sans SOMPK. La différence est statistiquement significative: (Z_cal_ = 9,23 > Z_th_ = 1,96; p <0,05). La courbe ménothermique biphasique a été retrouvée chez 48 patientes avec SOMPK (16%) versus 282 (94%) des patientes sans SOMPK. La différence est hautement significative:(Z_cal_ =30,9 > Z_th_ =1,96; p < 0,05). L’échographie pelvienne a révélé, chez toutes les patientes avec SOMPK, l'existence de microkystes reparties en périphérie, en moyenne de 12,37 ± 1,43 (extrême: 10 et 17) pour l'ovaire droit et 12,66 ± 1,43 (extrême: 10 et 16) pour l'ovaire gauche. Les dimensions ovariennes moyennes des ovaires des patientes avec SOMPK sont statistiquement supérieures à celles des patientes sans SOMPK P < 0,05 (Tableau 1). La proportion des patientes avec SOMPK qui accumulent 2000 à 2200 kcal/Jr est de 24,7% versus 34,7% des patientes sans SOMPK. Tandis que la fréquence de patientes avec SOMPK qui accumulent l’énergie de 2200 à 2400 kcal/jr est de 16,3% versus 28,7% des patientes sans SOMPK. La proportion des patientes qui accumulent au-delà de 2400 Kcal/Jr est de 59% versus 36,7% des patientes sans SOMPK. La moyenne d’énergie journalière accumulée chez les patientes avec SOMPK est de 2393 ± 766,4 kcal versus 2070, 55 ± 695 kcal. La différence est statistiquement significative (p < 0,05). Le risque de contracter le SOMPK, en présence d'une accumulation de plus de 2400 kcal/jour, est de 1,57 fois (RR = 1,57) (Tableau 2). La proportion des patientes avec SOMPK de niveau socio-économique élevé représente 169 (56,33%) patientes versus 64 (21,33%) patientes sans SOMPK P< 0,05. Elle est suivie de celle des niveaux moyen et bas respectivement 116 (38,67%) et 15 (5%) patientes. La proportion des patientes sans SOMPK avec un niveau de vie moyen, bas représente respectivement 124 (41,33), 112 (37%) des patientes. Le risque d'apparition de SOMPK est 2,03 fois plus élevé chez les patientes de niveau socio-économique élevé. La différence est hautement significative (p = 0,00) (Tableau 3). La proportion des patientes avec SOMPK exerçant les activités légères est de 43,7% versus une proportion de 9,3% dans le groupe des patientes sans SOMPK. La différence est significative (p < 0,05). Tandis que celle des patientes avec SOMPK exerçant des activités physiques modérées est de 49,3% contre 66,7% des patientes sans SOMPK. Par ailleurs la proportion des patientes avec SOMPK exerçant des activités intenses est de 7% contre 24% des patientes sans SOMPK. Le risque est de 2,15 fois plus élevé de développer le SOMPK chez les patientes exerçant des activités légères (p = 0,00). La proportion des patientes avec SOMPK consommant un ou deux repas par jour est faible soit respectivement 17 (5,66%),44 (14,67%) contre celle des patientes sans SOMPK soit respectivement 86 (28,66%), 87 (29%) P < 0,05. Nous avons noté, par ailleurs, que la proportion des patientes avec SOMPK qui consommaient 4 repas par jours est prépondérante soit 159 (53%) versus celle des patientes sans SOMPK soit 55 (18,34%) P <0,05. La proportion des patientes consommant 3 repas était similaire dans les 2 groupes de patientes soit 80 (26,67%) patientes avec SOMPK versus 72 (24%) patientes sans SOMPK.

**Tableau 1:** Les dimensions ovariennes moyennes (en millimètre) et les caractéristiques échographiques

Caractéristiques	Ovaire droit			Ovaire gauche		
	Patientes avec SOMPK n = 300	Patientes sans SOMPK n = 300		Patientes avec SOMPK n = 300	Patientes sans SOMPK n = 300	
	Moyenne ± ET	Moyenne ± ET	P	Moyenne ± ET	Moyenne ± ET	P
Petit axe de l'ovaire	4,22±0,26	2,29±0,38	P <0,05	4,18±0,24	2,35±0,97	P <0,05
Grand axe de l'ovaire	5,72 ± 0,27	3,79 ± 0,49	P <0,05	5,64 ± 0,36	3,89 ± 2,7	P <0,05
Profondeur de l'ovaire	1,87±0,40	1,86±1,10	P <0,05	1,87±0,46	1,87±0,89	P <0,05
Surface de l'ovaire	19,31 ± 2,10	6,94 ± 2,05	P <0,05	18,87 ± 2,27	7,32 ± 2,07	P <0,05
Volume	18,93 ± 2,02	6,80 ± 2,01	P <0,05	18,50 ± 2,22	7,17 ± 1,40	P <0,05
Microkystes/ovaire	12,37 ± 1,43	-		12,66 ± 1,43	-	

**Tableau 2:** Répartition des patientes selon l’énergie journalière accumulée par patiente

Energie (kcal)	Patientes avec SOMPK		Patientes sans SOMPK		RR	IC	p
	n = 300	%	n = 300	%			
2000 -2200	74	24,7%	104	34,70%	0,78	0,64 – 0,94	0,01
2200– 2400	49	16,3%	86	28,70%	0,67	0,53 – 0,85	0,00
>2400	177	59%	110	36,70%	1,57	1,33 – 1,85	0,00
Moyenne	2393±766,4 Kcal		2070, 55±695 Kcal				P < 0,05

Zcal= 9,23 >Zth = 1,96; P < 0,05

**Tableau 3:** Répartition des patientes selon le niveau socio – économique (Traissac et al, 1997)

Niveau socio- economique	Patientes avec SOMPK		Patientes sans SOMPK				
	n = 300	%	n = 300	%	RR	IC à 95%	p
Bas	15	5	112	37,33	0,20	0,12 – 1,32	0,00
Moyen	116	38,67	124	41,33	0,95	0,81 – 1,12	0,55
Elevé	169	56,33	64	21,33	2,03	1,73 - 2,38	0,00

## Discussion

Notre étude avait pour objectif de déterminer la prévalence de SOMPK dans les centres hospitaliers de Mbuji-Mayi et rechercher ses relations éventuelles avec certains facteurs nutritionnels et sociodémographiques. Les points saillants de l’étude indiquent que la prévalence de SOMPK est de 23,5% et les facteurs de risque identifiés sont la nulliparité, l'activité physique légère, le niveau socio-économique élevé et l'accumulation de plus de 2400 kcal. La prévalence de SOMPK rapportée pour l'ensemble des patientes reçues à la consultation gynécologique de quatre centres hospitaliers de Mbuji-Mayi est proche de l'observation faite par Clayton et coll. en 1992 qui ont observé une prévalence de 22%. En Europe, les fréquences de SOMPK ont été estimées entre 5% et 10%[14–16]. Les raisons de cette divergence ne sont pas clairement élucidées. Elles peuvent relever des facteurs ethniques, géographiques, épidémiologiques, nutritionnels, culturels ou de critères de diagnostic de SOMPK, caractéristiques de diverses régions d'autant plus que la définition de SOMPK varie selon les équipes. L’équipe américaine se base sur l’échographie, et l'Europe sur les critères de Rotterdam pour reconnaître le syndrome des ovaires micropolykystiques [9]. Par rapport à la parité, la proportion des patientes nullipares court un risque de 1,61 fois de contracter ce syndrome. Cette observation corrobore avec les études épidémiologiques qui constatent que les femmes nullipares avec SOMPK sont prédominantes [17, 18].

Notre étude indique que l'infertilité constitue le principal motif de consultation retrouvé chez 59% des patientes avec SOMPK versus 30% des patientes sans SOMPK. Goldzieher et Axelrod, dans une étude de 1963 regroupant 1079 cas provenant 167 publications, ont constaté que 74% des patientes consultaient pour l'infertilité [10]. Mboloko et coll. en 2004 ont noté en 2004 que 47,6% des patientes avec SOMPK versus 26,3% sans SOMPK consultaient pour l'infertilité [16]. En 2008, Goldenberg et Coll. ont trouvé que 75% des femmes touchées par le SOMPK consultaient pour l'infertilité. La cause de cette divergence n'est pas évidente. Elle peut relever des facteurs culturels et épidémiologiques [7, 8, 19]. Les principaux signes constatés à l'examen physique chez les patientes avec SOMPK demeurent l'hirsutisme 69%, l'acné 26%, acanthosis negrans 2,6%. Tozzini a observé, en 1995 que l'hirsutisme était le principal signe avec 54% de cas [20].

Notre étude a noté que l’énergie accumulée moyenne est de 2393 ± 766 Kcal chez les patientes avec SOMPK contre 2070, 55 ± 695 Kcal chez les patientes sans SOMPK. Le risque d'apparition de SOMPK est de 1,57 fois plus élevé chez les patientes ayant accumulé plus de 2400 kcal par rapport aux autres proportions des patientes ayant accumulé moins de 2400 kcal. La différence est statistiquement significative (p < 0,00). En France, l’étude individuelle nationale de consommation alimentaire des français mené en 2007 a indiqué que l'apport calorique journalier reste stable et se maintient à 1923 kcal [21]. Dans notre série, nous avons constaté que 43,7% des patientes avec SOMPK exercent des activités légères et 49,3% exercent les activités physiques modérées et seulement 7% les activités physiques intenses.

Erlichman et al [22] et Ruiz et al [13] ont montré qu'il existe un rapport évident entre l'activité physique intense et la stabilité pondérale et que, pour perdre le poids il faut exercer une activité physique intense [13, 22, 23]. De leur côté, Ekelund et coll. ont montré que des personnes pratiquant les activités physiques modérées de plus de 40 minutes par jour ont moins des tissus adipeux [24]. D'autres études indiquent que pour éviter de prendre le poids, il faut pratiquer une activité physique modérée c'est-à-dire rester debout ou marcher comme le font les femmes au foyer, les vendeurs, les serveurs de restaurant, les mécaniciens ou les commerçants. L'organisation mondiale de la santé recommande de pratiquer 30 minutes d'activité physique modérée presque tous les jours de la semaine pour un bon état de santé [25].

## Conclusion

Nos résultats ont montré une prévalence de 23,5% de SOMPK et une association entre le SOMPK avec la nulliparité; l’énergie emmagasinée de plus de 2400 Kcal, le niveau socio-économique élevé, l'activité physique légère. Des études ultérieures s'avèrent nécessaires pour mieux évaluer l'ampleur du risque observé.

### Limite de l’étude

Le poids des aliments consommés par les malades n'ont pas été préalablement pesé. Mais avant de calculer l’énergie des aliments consommés, nous avons payé les mêmes types d'aliments que les malades ont déclarés avoir mangé, et que nous avons pesés afin de reconstituer le poids des quantités de ces aliments consommées.
